# Drugs that reduce transmission of falciparum malaria

**DOI:** 10.1016/S1473-3099(18)30070-7

**Published:** 2018-02-06

**Authors:** Ric N Price, Nicholas J White

**Affiliations:** Centre for Tropical Medicine and Global Health, Nuffield Department of Clinical Medicine, University of Oxford, Oxford OX3 7FZ, UK; Global Health Division, Menzies School of Health Research, Charles Darwin University, Darwin, NT, Australia; Centre for Tropical Medicine and Global Health, Nuffield Department of Clinical Medicine, University of Oxford, Oxford OX3 7FZ, UK; Mahidol Oxford Tropical Medicine Research Unit, Faculty of Tropical Medicine, Mahidol University, Bangkok, Thailand

Substantial gains have been made in reducing the global burden of malaria, much of which can be attributed to greater access to prompt diagnosis and highly effective treatment. However, as endemic countries commit to eliminating malaria, more aggressive interventions are needed to target the large number of apparently healthy individuals who harbour transmissible malaria parasites. Although most national antimalarial guidelines recommend artemisinin combination therapy for the management of uncomplicated falciparum malaria, chemopreventive strategies have generally adopted non-artemisinin combination therapy regimens such as sulfadoxine-pyrimethamine and amodiaquine. Artemisinin and its derivatives reduce carriage of sexual stages of the malaria parasites (gametocytes) that are infectious to the mosquito vector, but neither artemisinin combination therapies nor sulfadoxine-pyrimethamine and amodiaquine prevent transmission from fully mature *Plasmodium falciparum* gametocytes that might be present at the time of treatment.

Primaquine has potent activity against mature *P falciparum* gametocytes. Although primaquine can induce haemolysis in individuals with glucose-6-phosphate dehydrogenase (G6PD) deficiency, a single low dose of 0·25 mg/kg is safe and well tolerated, even in G6PD-deficient individuals, and highly effective in reducing transmissibility.^1^,^2^ Methylene blue also has potent gametocytocidal activity.^3^ Its antimalarial properties in vivo were first reported by Paul Ehrlich in the 1890s.^4^ However, despite the ex-vivo activity of methylene blue against many plasmodium stages and species, when given alone its clinical efficacy is insufficient and has been hampered by poor tolerability, including gastrointestinal side effects and discolouration of skin and urine.

In *The Lancet Infectious Diseases*, Alassane Dicko and colleagues^5^ report the results of a phase 2, single-blind, randomised controlled trial in Mali that assessed the effects of antimalarial drugs on *P falciparum* transmissibility. 40 G6PD-normal asymptomatic Malian participants were randomly assigned to receive either sulfadoxine-pyrimethamine and amodiaquine (the currently recommended seasonal malaria chemopreventive for administration to children living in the high seasonal transmission belt across the Sahel), with or without primaquine (0·25 mg/kg single dose as recommended in low transmission settings to reduce *P falciparum* transmissibility). Another 40 participants were randomly assigned to receive dihydroartemisinin-piperaquine with or without methylene blue (15 mg/kg per day for 3 days). Transmissibility was assessed by molecular quantification of sexual stage-specific mRNAs and by membrane feeding blood to mosquitos and counting the oocytes that formed. Both primaquine and methylene blue were highly effective in reducing gametocytaemia and preventing transmissibility within 2 days of starting treatment.

This small yet detailed study^5^ supports the excellent efficacy of primaquine and confirms that methylene blue is also a potent *P falciparum* gametocytocidal drug in vivo, as suggested by earlier studies. Although the study population was limited to male participants who were G6PD-normal, previous studies have shown that a single low dose of primaquine (0·25 mg/kg) is safe in people with moderate severity G6PD deficiency (G6PD-Mahidol), and that a 3-day regimen of methylene blue was also safe in the generally less severe G6PD A-variant prevalent in Africa.^6^,^7^

A single low dose of primaquine is easy to administer, safe, efficacious, and inexpensive. So is there need for further exploration of an alternative gametocytocidal agent? Reliance on a single therapeutic intervention to reduce mosquito infectivity is risky. Artemisinin-resistant *P falciparum* has emerged in the Greater Mekong subregion and is spreading.^8^ In affected areas, patients treated with artemisinin-based combination therapy take longer to clear their peripheral parasitaemia and are at greater risk of having patent gametocytaemia and failing treatment, all of which fuel the spread of resistance both to artemisinin and its partner drugs.^9^ WHO’s global plan for containing artemisinin resistance recommends adding a single dose of primaquine to reduce ongoing transmission.^10^ This study^5^ suggests that methylene blue is a potential alternative gametocytocidal drug. It retains potent ex-vivo activity against multidrug resistant *P falciparum*^11^ and when combined with artesunate in sub-Saharan Africa, it achieved faster parasite clearance compared to artesunate-amodiaquine alone.^3^,^12^ However, further clinical trials are needed to optimise dosing and confirm these potential benefits in patients with artemisinin-resistant *P falciparum*.
